# Effects of hawthorn seed oil on plasma cholesterol and gut microbiota

**DOI:** 10.1186/s12986-022-00690-4

**Published:** 2022-08-12

**Authors:** Erika Kwek, Chi Yan, Huafang Ding, Wangjun Hao, Zouyan He, Jianhui Liu, Ka Ying Ma, Hanyue Zhu, Zhen-Yu Chen

**Affiliations:** 1 School of Life Sciences, The Chinese University of Hong Kong, Shatin, Hong Kong China; 2 School of Public Health, Guangxi Medical University, Nanning, 530021 China; 3 College of Food Science and Engineering, Nanjing University of Finance and Economics, Nanjing, 210023 China; 4 School of Food Science and Engineering / South China Food Safety Research Center, Foshan University, Foshan, Guangdong China

**Keywords:** Hawthorn seed oil, Cardiovascular disease, Hypercholesterolemia, Gut microbiota

## Abstract

**Background:**

Hypercholesterolemia and gut microbiota dysbiosis are associated with the risk of cardiovascular diseases. Hawthorn fruits has shown to be cardioprotective and hypocholesterolemic. However, no studies to date have studied the biological activity of hawthorn seed oil (HSO). The present study was to investigate if HSO could favourably reduce plasma cholesterol and modulate gut microbiota in hypercholesterolemia hamsters.

**Methods:**

Golden Syrian hamsters (age, 8 weeks) were randomly divided into five groups (*n* = 8, each) and fed one of the following five diets, namely a non-cholesterol diet, a high cholesterol diet containing 0.15% cholesterol (HCD); a HCD diet with addition of 4.75% HSO (LHSO), a HCD diet with addition of 9.5% HSO (HHSO), a HCD diet with addition of 0.50% cholestyramine as positive control diet. After 6-week dietary intervention, plasma lipids, inflammatory markers, atherosclerosis plaque, hepatic and fecal lipids were quantified. Microbiota in fresh feces were analysed by sequencing 16S rRNA genes, while RT-PCR and Western blot analyses were employed to quantify the expression of genes involved in cholesterol homeostasis.

**Results:**

HSO at a dose of 9.5% HSO could decrease plasma cholesterol and non-HDL-cholesterol by 15%. Additionally, both HSO experimental groups also suppressed mRNA of 3-Hydroxy-3-Methylglutaryl-CoA Reductase (HMG-CoA-R). Supplementation of HSO at 4.75% could significantly increase the excretion of fecal acidic sterols, accompanied by elevation of short-chain fatty acid levels in feces. The analyses of gut microbiome indicated that HSO supplementation could selectively alter the genera abundance of gut bacteria that were correlated with cholesterol metabolism including *unclassified_f__Christensenellaceae, Ruminococcaceae_NK4A214_ group, norank_o_Gastranaerophilales, Faecalibaculum, Peptococcus, norank_f__Clostridiales_vadinBB60_group* and *Ruminococcus_2.*

**Conclusions:**

HSO supplementation was able to decrease plasma cholesterol by favourably modulating gut microbiota composition and gut-derived metabolites associated with cholesterol regulation.

**Graphical Abstract:**

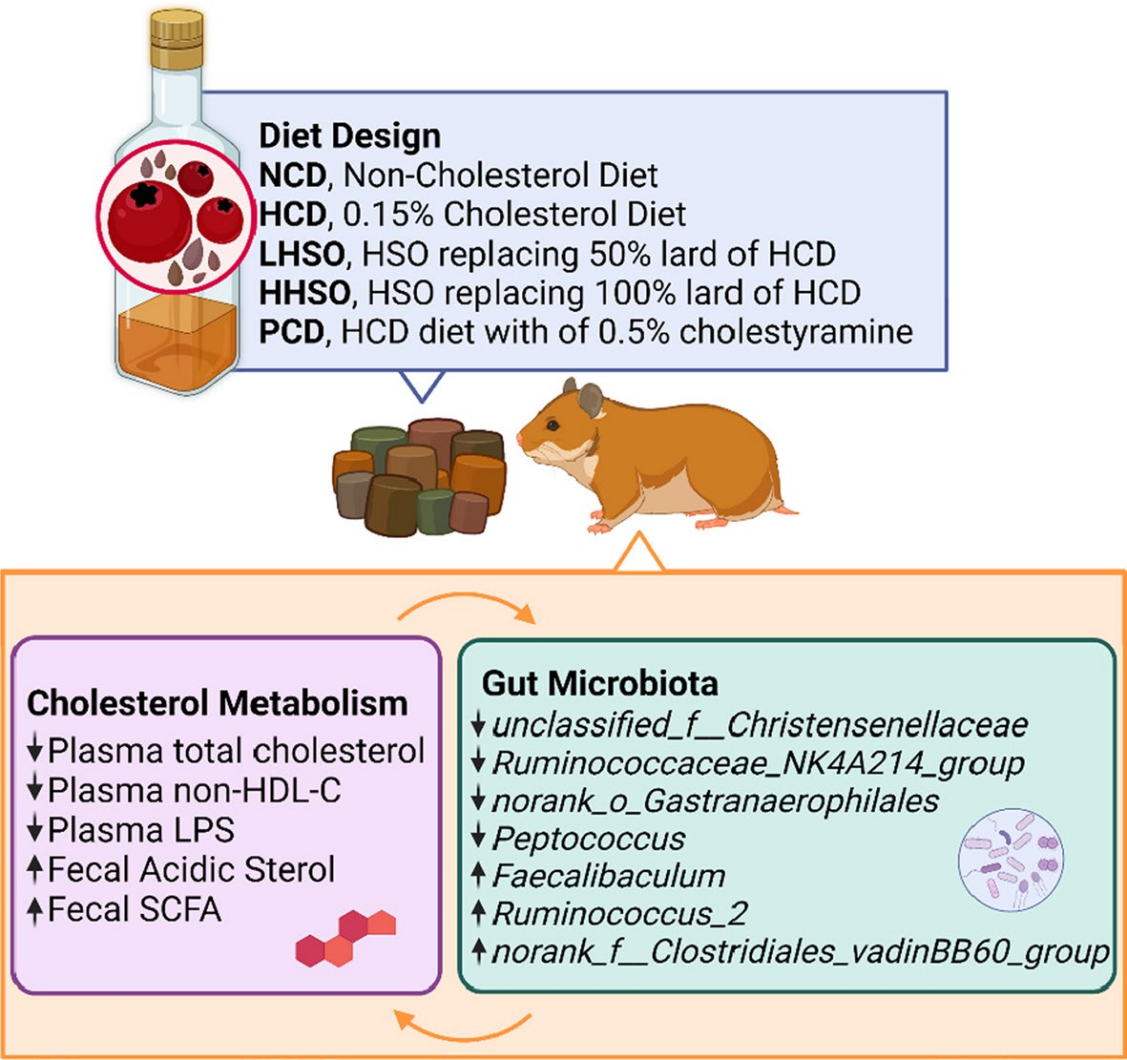

## Background

Hypercholesterolemia is one of the major risk factors contributing to the development of cardiovascular disease (CVD) [1–5]. Recent studies have found that gut microbiota dysbiosis and gut-derived adverse metabolites can contribute to the onset of CVD [6, 7]. Functional foods and supplements of nutraceuticals have shown to be effective in regulating plasma cholesterol levels [8–10]. In addition, the increasing evidence has demonstrated that macronutrients such as polyunsaturated fatty acids (PUFAs) can modulate the gut microbiota across various animal models [11–13].

Hawthorn (*Crataegus* spp.) is a member of the Rosaceae family that originates from Northern Asia, Europe and North America. Most studies regarding cardioprotective benefits of hawthorn have focused on its fruits, leaves, flowers and roots [14–18]. Extracts from different parts of hawthorn plant have been reported to possess anti-atherosclerotic activity and suppress the cholesterol synthesis [17, 19–21]. However, little is known about hawthorn seed oil (HSO) and its effects on plasma cholesterol and gut microbiota. The present study was therefore designed to investigate if hawthorn seed oil could favourably modulate cholesterol metabolism and gut microbiota in hypercholesterolemic hamsters.

## Materials and methods

### HSO and diet preparation

HSO was obtained from Xi’an Tuofeng Biotechnology Co., Ltd (Xi’an City, Shaanxi Province, China). Five diets were prepared based on AIN-93 diet as previously described with minor modification [22, 23]. First, a non-cholesterol diet (NCD) was formulated with the following components (g/kg diet): corn starch (484), casein (230), sucrose (113), lard (95), mineral mix (38), vitamin mix (19), gelatin (19), and DL-Methionine (1). A high cholesterol diet (HCD) was similarly prepared as NCD except 0.15% cholesterol (w/w) was added. A low HSO diet (LHSO) was prepared by substituting half the amount of lard (47.5 g) in HCD diet. A high HSO diet (HHSO) was prepared by complete replacement of lard (95 g) in HCD diet. Finally, a positive control diet (PCD) was prepared by adding 0.5% cholestyramine into HCD (Table 1). Cholestyramine is a medicine which can effectively reduce plasma cholesterol when administered at 0.5% dosage, as supported by previous studies [22, 24].

**Table 1:** Composition of five diets

	NCD	HCD	LHSO	HHSO	PCD
Ingredients (g kg^−1^ diet)					
Corn starch	484	484	484	484	484
Casein	230	230	230	230	230
Sucrose	113	113	113	113	113
Lard	95	95	47.5	–	95
Hawthorn seed oil	–	–	47.5	95	–
Mineral mix	38	38	38	38	38
Vitamin mix	19	19	19	19	19
Gelatin	19	19	19	19	19
DL-methionine	1	1	1	1	1
Cholesterol (0.15%)	–	1.5	1.5	1.5	1.5
Cholestyramine	–	–	–	–	5

NCD, Non-cholesterol diet; HCD, 0.15% Cholesterol diet; LHSO, Hawthorn seed oil replacing 50% lard of HCD; HHSO, Hawthorn seed oil replacing 100% lard of HCD; PCD, HCD diet with addition of 0.5% cholestyramine

### Animals and study design

Male 8-week-old Golden Syrian hamsters (*Mesocricetus auratus*) were randomly divided into five groups (*n* = 8 each) and fed one of five diets described above. All animals were kept under a regulated condition (12–12 h rotating light–dark cycle at 23 °C) with free access to tap water. Feeding was conducted for a period of 6 weeks, with weekly measurement of body weights and diet intake. Blood collection from the retro-orbital sinus under light anaesthesia was performed after overnight fasting at week 0 and week 6. Total fecal outputs from week 6 were collected followed by being freeze-dried and ground before storage in a − 80 °C freezer. Fresh fecal samples used for gut microbial analyses through 16S rRNA sequencing was collected under sterile environment as previously described [25]. At the end of week 6, hamsters were sacrificed using carbon dioxide as anaesthetic. Organs were collected and stored at − 80 °C. The entire experimental protocol was approved by the Animal Ethics Experimentation Committee, of The Chinese University of Hong Kong (Ref. No.: 21-169-MIS).

### Fatty acid composition of HSO and lard

Fatty acids in HSO and lard were quantified using gas chromatography (GC), as previously described [11, 26]. Briefly, the fatty acid methyl esters (FAMEs) were firstly prepared and analysed on a HP Innowax capillary column (Agilent Technologies, Santa Clara, CA, USA) in Shimadzu Gas Chromatograph 2010 (Shimadzu, Kyoto, Japan). Individual fatty acid methyl esters were quantified according to the amount of internal standard heptadecanoic acid added into the sample.

### Phytosterol analysis

Phytosterols in HSO were quantified using a gas chromatographic method as previously described [27]. In brief, HSO was firstly saponified, phytosterols were extracted and converted to their corresponding TMS derivatives followed by separation on a SAC™-5 capillary column (Supelco, Bellefonte, PA, USA) in Shimadzu GC-2010 (Shimadzu, Kyoto, Japan). Phytosterols were quantified according to the amount of internal standard (5α-cholestane) added into HSO prior to the analysis.

### Plasma lipid analysis

Plasma total cholesterol (TC), triacylglycerols (TG) and high-density lipoprotein cholesterol (HDL-C) were quantified using respective commercial kits (Stanbio Laboratories, Boerne, Texas, USA). Non-HDL-C (nHDL-C) level was calculated as the value obtained after subtracting HDL-C from total plasma TC.

### Measurement of atherosclerotic plaque area

The total atherosclerotic plaque are were quantified as previously described [28]. Aorta samples were firstly prepared by removing surrounding fat and tissues. Following dehydration with propan-2-ol, each aorta was stained with Oil Red O. The ImageJ software (NIH, LOCI, USA) was used to analyse the percentage of atherosclerotic plaques.

### Liver cholesterol analysis

The total lipids from the liver sample were firstly extracted and saponified. The liver cholesterol was converted to its corresponding TMS derivative as previously stated [22, 28]. The cholesterol-TMS derivative was then analysed using a Shimadzu GC-2010 Chromatography fitted with a column SAC™-5 column (Supelco, Bellefonte, PA, USA). 5α-Cholestane was used as an internal standard for the quantification of cholesterol.

### Liver lipid analysis

Total lipids in the form of total fatty acids were quantified as previously described [27]. In brief, total lipids were firstly extracted and converted to the corresponding FAMEs. Afterward, the individual FAMEs were separated and quantified in a Shimadzu GC-2010 fitted with a HP-Innowax capillary column (Agilent Technologies, Santa Clara, CA, USA); heptadecanoic acid was used as an internal standard.

### Fecal neutral and acidic sterol analyses

Total and individual neutral and acidic sterols in feces were analysed as previously described [27]. In brief, the fecal samples were saponified and total neutral sterols were extracted into cyclohexane. Subsequently, diethyl ether was used to extract the total acidic sterols. Afterward, neutral and acidic sterols were converted to their corresponding TMS derivatives. A SAC™-5 capillary column (Supelco, Bellefonte, PA, USA) was used to separate and quantify the individual neutral and acidic sterols. 5α-cholestane was an internal standard for neutral sterols, and hyodeoxycholic acid was an internal standard for acidic sterols.

### Short-chain fatty acid (SCFA) analysis

Fecal SCFAs were analysed using a Shimadzu GC-2010 equipped with a CP-FFAP CB capillary column (Agilent Technologies, CA, USA). Ethanol solution (50%) was used for consecutive sonication and extractions of SCFAs. 2-Ethylbutyric acid was used as the internal standard [29].

### Plasma lipopolysaccharide (LPS) analysis

An LPS-specific commercial kit (Cusabio, Wuhan, China) was used to quantify the concentration of LPS in plasma using enzyme-linked immunosorbent assay technique. Accordingly, a standard curve was firstly constructed, the absorbance of plasma was measured, and LPS was calculated based on the standard curve.

### Real-time PCR (RT-PCR) and western blot

The following genes related to cholesterol metabolism and absorption were measured at both transcriptional and translational levels: Hepatic LDL receptor (LDLR), Liver X receptor-alpha (LXRα), sterol regulatory element-binding protein 2 (SREBP2), cholesterol-7α-hydroxylase (CYP7A1), and 3-Hydroxy-3-Methylglutaryl-CoA Reductase (HMG-CoA-R). Targets for intestinal samples include: Acetyl-CoA Acetyltransferase 2 (ACAT2), Niemann-pick C1-Like 1 (NPC1L1), microsomal triacylglycerol transport protein (MTP), and ATP binding cassette subfamily G -5 (ABCG5) and -8 (ABCG8). As per previous method, cDNA was converted from extracted RNA for RT-PCR analysis [22]. Quantification of RT-PCR was conducted according to comparative-threshold method using StepOnePlus RT-PCR System to measure mRNA expression.

Western blotting was conducted for protein mass analysis accordingly [22]. In brief, sample preparation included extraction and concentration measurement using BCA Assay. Denatured proteins were separated according to molecular weight, using 10% sodium dodecyl sulfate polyacrylamide gel electrophoresis (SDS-PAGE). Polyvinylidene difluoride membrane containing transferred proteins are blocked by 3% bovine serum albumin in Tris-buffered saline with Tween-20 (TBST). Membranes are rotated overnight in specific primary antibodies at 4 °C. After binding with secondary antibodies and washing with TBST, the membranes are exposed to chemiluminescence using enhanced chemiluminescence (ECL) agents. β-Actin was chosen as a house-keeping gene for all normalization.

### Gene sequencing of 16S ribosomal RNA

Microbiota in fresh feces were analysed by sequencing 16S rRNA genes. As described in previous procedures, V3-V4 section of the rRNA genes were targeted using Illumina MiSeq (San Diego, CA, USA) [29]. Clustering of operational taxonomic units (OTUs) were conducted at 97% similarity threshold, and taxonomy was obtained via Silva database. Rarefaction curves were constructed for α-diversity analysis of community—Sobs and Shannon index. Principal coordinates analysis (PCoA) was used to represent β-diversity. Threshold of 2.5 was selected to determine altered genera using linear discriminant analysis (LDA) Effect Size (LEfSe) analysis. Wilcoxon rank-sum test was selected for differentiating changes to genus abundance. Using Spearman’s correlation, the environmental parameters alongside genera changes were also analysed [29].

### Statistical analysis

SPSS Statistics (SPSS Inc., v. 15.0) was used to analyse statistical significance (*p* < 0.05) between among the five groups. One-way analysis of variance (ANOVA) and least significant difference (LSD) post-hoc test was conducted. Values were presented as Mean ± Standard Deviation (SD). Statistical analysis on gut microbiota-related data were performed using bioinformatic platform of Majorbio ISanger Cloud (www.i-sanger.com).

## Results

### Fatty acid and phytosterol composition of HSO

HSO contained a high amount of PUFAs with linoleic acid (18:2n-6) accounting for 58.84% and α-linolenic acid (18:3n-3) accounting for 4.77%. HSO was also rich in monounsaturated fatty acid oleic acid (18:1n-9, 24.90%). Comparatively, lard was more abundant in saturated fatty acids namely palmitic acid (16:0) and stearic acid (18:0). HSO also contained 205 mg/100 g phytosterols in an order of β-sitosterol > stigmasterol > campesterol > Δ7-stigmastenol > stigmastanol, citrostadienol > Δ7-avenasterol > ergosterol (Table 2).

**Table 2:** Composition of fatty acids and phytosterols in hawthorn seed oil (HSO) and lard

	HSO	Lard
(A) Fatty acids (%)		
16:0	10.86 ± 0.02	27.20 ± 0.02
18:0	3.45 ± 0.00	10.26 ± 0.02
*Total SFAs*	14.31 ± 0.03	44.71 ± 0.01
18:1n-9	24.90 ± 0.03	38.46 ± 0.01
*Total MUFAs*	24.90 ± 0.03	38.46 ± 0.01
18:2n-6	50.84 ± 0.01	10.26 ± 0.02
18:3n-3	4.77 ± 0.01	0.71 ± 0.00
*Total PUFAs*	55.61 ± 0.01	10.97 ± 0.02
(B) Phytosterols (mg/100 g)		
Ergosterol	1.04 ± 0.09	N.D
Campesterol	32.53 ± 0.16	N.D
Stigmasterol	33.50 ± 0.24	N.D
β-sitosterol	105.50 ± 1.36	N.D
Stigmastanol	9.74 ± 0.73	N.D
Δ7-stigmastenol	10.95 ± 1.69	N.D
Δ7-avenasterol	3.78 ± 0.10	N.D
Citrostadienol	8.16 ± 1.24	N.D
*Total*	205.20 ± 34.52	N.D

Values are expressed as Mean ± S.D

N.D., Non-detectable; SFAs, Saturated fatty acids; MUFAs, Monounsaturated fatty acids; PUFAs, Polyunsaturated fatty acids

### Food intake, body, and organ weights

During the 6-week feeding, no significant differences were observed in food intake and body weights among the five groups of hamsters. Relative organ weights of LHSO, HHSO and HCD were statistically comparable (Table 3).

**Table 3:** Daily food intake, changes in body weights, and relative organ weights in hamsters fed one of the five diets

	NCD	HCD	LHSO	HHSO	PCD	*p* value
Daily food intake (g/d)	8 ± 0	9 ± 1	9 ± 1	9 ± 1	9 ± 1	0.95
Body weight (g)						
Initial	115 ± 9	114 ± 7	114 ± 9	115 ± 7	117 ± 10	0.96
Final	133 ± 13	128 ± 11	133 ± 12	134 ± 10	137 ± 16	0.77
Relative organ weight (% body weight)						
Heart	0.4 ± 0.0^a^	0.3 ± 0.0^b^	0.3 ± 0.0^b^	0.3 ± 0.0^b^	0.3 ± 0.0^ab^	0.01
Testis	3.1 ± 0.3	2.6 ± 0.7	2.4 ± 0.7	2.9 ± 0.3	2.2 ± 0.8	0.08
Kidney	0.8 ± 0.2	0.8 ± 0.0	0.8 ± 0.1	0.7 ± 0.0	0.8 ± 0.1	0.99
Perirenal adipose tissue	1.5 ± 0.5	1.5 ± 0.3	1.4 ± 0.2	1.5 ± 0.2	1.5 ± 0.2	0.91
Epididymal adipose tissue	2.1 ± 0.6	1.8 ± 0.3	1.6 ± 0.1	1.9 ± 0.3	1.9 ± 0.4	0.28
Liver	4.9 ± 0.7^bc^	5.7 ± 0.6^a^	5.2 ± 0.4^ab^	5.3 ± 0.3^ab^	4.6 ± 0.3^c^	< 0.01

NCD, Non-cholesterol diet; HCD, 0.15% Cholesterol diet; LHSO, Hawthorn seed oil substituting 50% lard; HHSO, Hawthorn seed oil substituting 100% lard, PCD, HCD with 0.5% cholestyramine

Data is expressed as mean ± S.D., where different letters in superscript indicate statistical difference, *p* < 0.05

### Effects of HSO on plasma lipid profile

Plasma TC, HDL-C, nHDL-C, and TG were similar among the five diet groups at week 0. After 6 weeks of feeding study, feeding HCD diet elevated TC concentration by 49% compared with that in NCD group. Feeding PCD diet suppressed HCD-induced increase of plasma TC by 35%. Feeding two experimental diets (LHSO and HHSO) reduced plasma TC significantly by 15–16% compared with HCD group. Additionally, two experimental diets could also lower nHDL-C level by 17–25%. Plasma TG in LHSO and HHSO groups showed a decreasing trend, however, they were not statistically different from HCD group (Table 4).

**Table 4:** Changes to plasma lipid profile in hamsters fed one of the five diets

	NCD	HCD	LHSO	HHSO	PCD
Week 0					
TC (mg/dL)	165.7 ± 27.2	163.5 ± 25.8	161.3 ± 15.2	167.8 ± 25.9	170.6 ± 21.5
HDL-C (mg/dL)	137.1 ± 23.9	144.0 ± 20.8	135.6 ± 20.8	132.0 ± 33.2	134.0 ± 19.6
nHDL-C (mg/dL)	28.5 ± 10.3	19.5 ± 24.9	25.7 ± 13.9	35.7 ± 19.5	36.5 ± 15.6
TG (mg/dL)	59.8 ± 17.2	61.3 ± 11.6	68.3 ± 12.1	63.1 ± 10.0	71.9 ± 15.1
nHDL-C/HDL-C	0.2 ± 0.0	0.1 ± 0.1	0.2 ± 0.1	0.3 ± 0.2	0.2 ± 0.1
HDL-C/TC	0.8 ± 0.0	0.8 ± 0.1	0.8 ± 0.0	0.7 ± 0.1	0.7 ± 0.0
Week 6					
TC (mg/dL)	160.2 ± 22.7^c^	238.8 ± 20.9^a^	200.6 ± 14.4^b^	202.1 ± 28.1^b^	155.4 ± 9.8^c^
HDL-C (mg/dL)	113.3 ± 15.1^c^	157.4 ± 16.4^a^	133.2 ± 11.1^b^	140.7 ± 18.4^b^	129.5 ± 3.9^bc^
nHDL-C (mg/dL)	46.8 ± 12.4^c^	81.4 ± 12.0^a^	67.3 ± 16.3^ab^	61.4 ± 15.9^bc^	25.8 ± 9.0^d^
TG (mg/dL)	97.5 ± 45.0^b^	184.6 ± 87.2^a^	150.3 ± 41.2^ab^	132.5 ± 47.4^ab^	121.9 ± 13.9^ab^
nHDL-C/HDL-C	0.4 ± 0.1^a^	0.5 ± 0.1^a^	0.5 ± 0.1^a^	0.4 ± 0.1^a^	0.2 ± 0.0^b^
HDL-C/TC	0.7 ± 0.0^b^	0.6 ± 0.0^b^	0.6 ± 0.0^b^	0.7 ± 0.0^b^	0.8 ± 0.0^a^

TC, Plasma total cholesterol; HDL-C, High density lipoprotein cholesterol, TG, Triacylglycerol; nHDL-C, Non-HDL cholesterol; NCD, Non-cholesterol diet; HCD, 0.15% Cholesterol diet; LHSO, Hawthorn seed oil substituting 50% lard; HHSO, Hawthorn seed oil substituting 100% lard; PCD, HCD with 0.5% cholestyramine

Data is expressed as mean ± S.D., where different letters in superscript indicate statistical difference, *p* < 0.05

### Effects of HSO on atherosclerotic plaque and plasma LPS

**Fig. 1:**
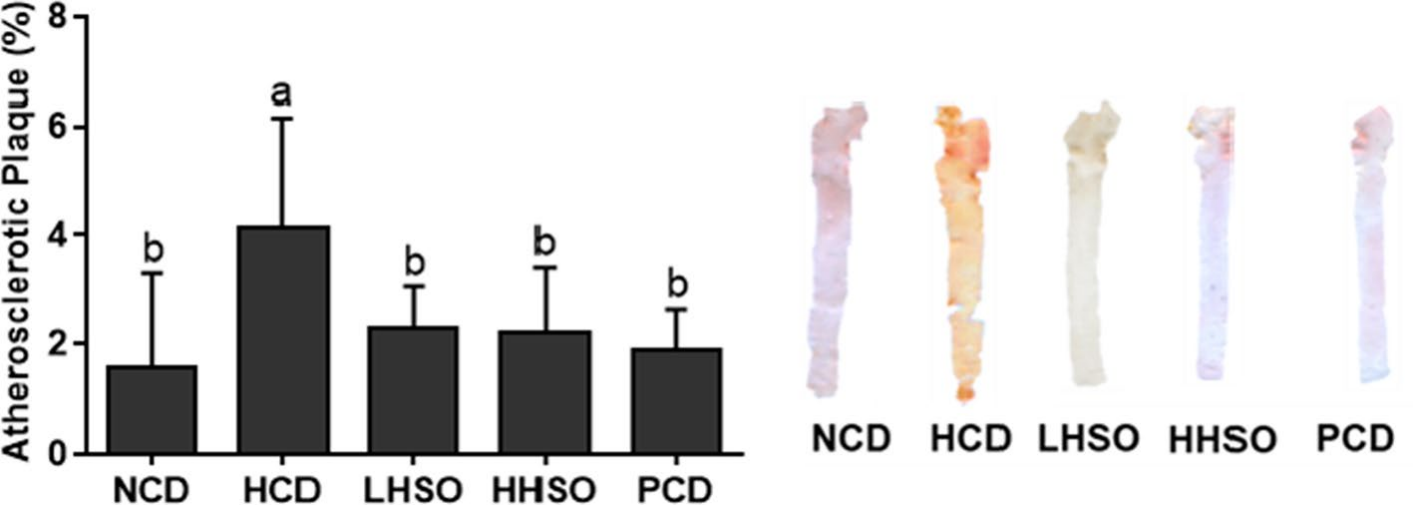
Atherosclerotic plaque area in hamsters fed one of the five diets: non-cholesterol diet (NCD); 0.15% cholesterol diet (HCD); hawthorn seed oil substituting 50% lard (LHSO); hawthorn seed oil substituting 100% lard (HHSO), and HCD with 0.5% cholestyramine (PCD). Data is expressed as mean ± S.D., where different letters in superscript indicate statistical difference, *p* < 0.05

**Fig. 2:**
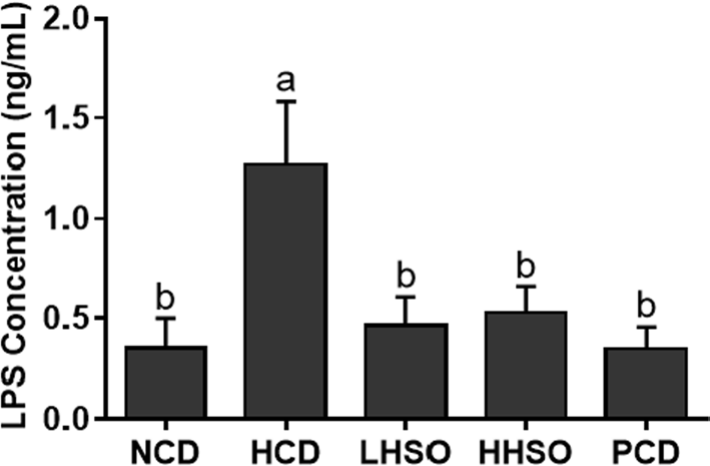
Changes in plasma lipopolysaccharide (LPS) concentration in hamsters fed one of the five diets: non-cholesterol diet (NCD); 0.15% cholesterol diet (HCD); hawthorn seed oil substituting 50% lard (LHSO); hawthorn seed oil substituting 100% lard (HHSO), and HCD with 0.5% cholestyramine (PCD). Data is expressed as mean ± S.D., where different letters in superscript indicate statistical difference, *p* < 0.05

Feeding HCD diet led to a 166% increase in atherosclerotic plaque development, compared with NCD group. Feeding PCD diet significantly decreased atherosclerotic accumulation by 55%, while feeding two HSO diets also prevented HCD-induced atherosclerotic plaque area by 44–46% (Fig. 1). Feeding HCD diet elevated plasma LPS concentration by 260% compared with feeding NCD diet. Compared to HCD, PCD group had a 72.5% decrease in LPS concentration. Similarly, HSO experimental groups significantly decreased plasma LPS by 58–63% (Fig. 2).

### Effects of HSO on liver lipid and cholesterol

**Fig. 3:**
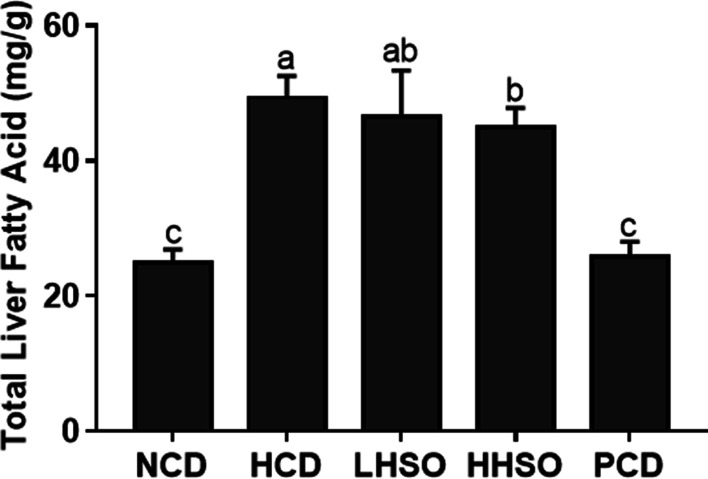
Liver fatty acid changes in hamsters fed one of the five diets: non-cholesterol diet (NCD); 0.15% cholesterol diet (HCD); hawthorn seed oil substituting 50% lard (LHSO); hawthorn seed oil substituting 100% lard (HHSO), and HCD with 0.5% cholestyramine (PCD). Data is expressed as mean ± S.D., where different letters in superscript indicate statistical difference, *p* < 0.05

**Fig. 4:**
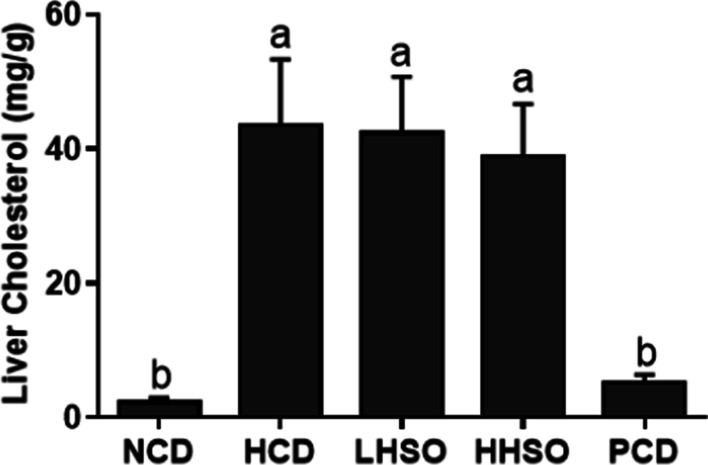
Changes in liver cholesterol accumulation in hamsters fed one of the five diets: non-cholesterol diet (NCD); 0.15% cholesterol diet (HCD); hawthorn seed oil substituting 50% lard (LHSO); hawthorn seed oil substituting 100% lard (HHSO), and HCD with 0.5% cholestyramine (PCD). Data is expressed as mean ± S.D., where different letters in superscript indicate statistical difference, *p* < 0.05

Feeding HCD diet increased liver lipids significantly by 98.5% compared with feeding NCD group. Feeding PCD diet could prevent the liver lipid accumulation by 48%. Feeding LHSO did not significantly affect liver lipids, whereas feeding HHSO significantly lowered the lipid accumulation by 9% compared with HCD group (HCD, 49.3; LHSO, 46.5; HHSO, 44.9 mg/g liver) (Fig. 3). The two experimental diets had no effect on liver cholesterol (Fig. 4). However, compared with HCD, PCD diet significantly lowered hepatic cholesterol levels (HCD, 43.5; PCD, 5.2 mg/g liver).

### Effects of HSO on fecal acidic and neutral sterols

Coprostanol, coprostanone and dihydrocholesterol are the main microbial metabolites of unabsorbed cholesterol [25]. After 6 weeks of feeding, the output of total fecal neutral sterols was not affected in LHSO group, whereas HHSO decreased its excretion relative to HCD (HCD, 1.3; LHSO, 1.17; HHSO, 1.05 mg/hamster/day). Fecal acidic sterols include primary bile acids—chenodeoxycholic acid (CDCA) and cholic acid (CA), as well as secondary bile acids—lithocholic acid (LCA) and deoxycholic acid (DCA). At the end of diet intervention, HHSO increased total fecal acidic sterols by 15%, although it did not reach statistical significance. LHSO feeding, however, was able to promote the excretion of acidic sterols significantly by 102% (HCD, 2.31; HHSO, 2.65; LHSO, 4.66 mg/hamster/day). Similarly, PCD also elevated the amounts of acidic sterols in feces (Table 5).

**Table 5:** Daily excretion of fecal neutral and acidic sterol in hamsters fed one of the five diets

	NCD	HCD	LHSO	HHSO	PCD
Neutral sterols (mg/hamster/day)					
Coprostanol	0.20 ± 0.09^d^	0.62 ± 0.17^b^	0.46 ± 0.12^bc^	0.38 ± 0.11^cd^	1.36 ± 0.29^a^
Coprostanone	0.01 ± 0.00^c^	0.01 ± 0.00^c^	0.01 ± 0.00^c^	0.02 ± 0.01^b^	0.04 ± 0.01^a^
Cholesterol	0.33 ± 0.07^c^	0.55 ± 0.10^a^	0.51 ± 0.11^ab^	0.44 ± 0.07^ac^	0.40 ± 0.18^bc^
Dihydrocholesterol	0.13 ± 0.02^c^	0.16 ± 0.02^bc^	0.19 ± 0.03^ab^	0.21 ± 0.03^a^	0.17 ± 0.04^b^
Total	0.66 ± 0.08^d^	1.33 ± 0.27^b^	1.17 ± 0.15^bc^	1.05 ± 0.08^c^	1.98 ± 0.15^a^
Acidic sterols (mg/hamster/day)					
Lithocholic acid	1.20 ± 0.34^c^	1.85 ± 0.69^c^	3.27 ± 1.02^b^	1.84 ± 0.57^c^	5.19 ± 1.54^a^
Deoxycholic acid	0.08 ± 0.04^b^	0.07 ± 0.02^b^	0.14 ± 0.07^b^	0.08 ± 0.02^b^	0.42 ± 0.16^a^
Chenodeoxycholic acid	0.33 ± 0.21^b^	0.19 ± 0.09^b^	0.69 ± 0.26^b^	0.38 ± 0.11^b^	5.04 ± 1.78^a^
Cholic acid	0.12 ± 0.04^c^	0.20 ± 0.08^bc^	0.57 ± 0.20^a^	0.35 ± 0.06^b^	0.59 ± 0.28^a^
Total	1.73 ± 0.52^c^	2.31 ± 0.84^c^	4.66 ± 1.36^b^	2.65 ± 0.73^c^	11.25 ± 3.64^a^

NCD, Non-cholesterol diet; HCD, 0.15% Cholesterol diet; LHSO, Hawthorn seed oil substituting 50% lard; HHSO, Hawthorn seed oil substituting 100% lard; PCD, HCD with 0.5% cholestyramine

Data is expressed as mean ± S.D., where different letters in superscript indicate statistical difference, *p* < 0.05

### Effects of HSO on fecal SCFAs

**Fig. 5:**
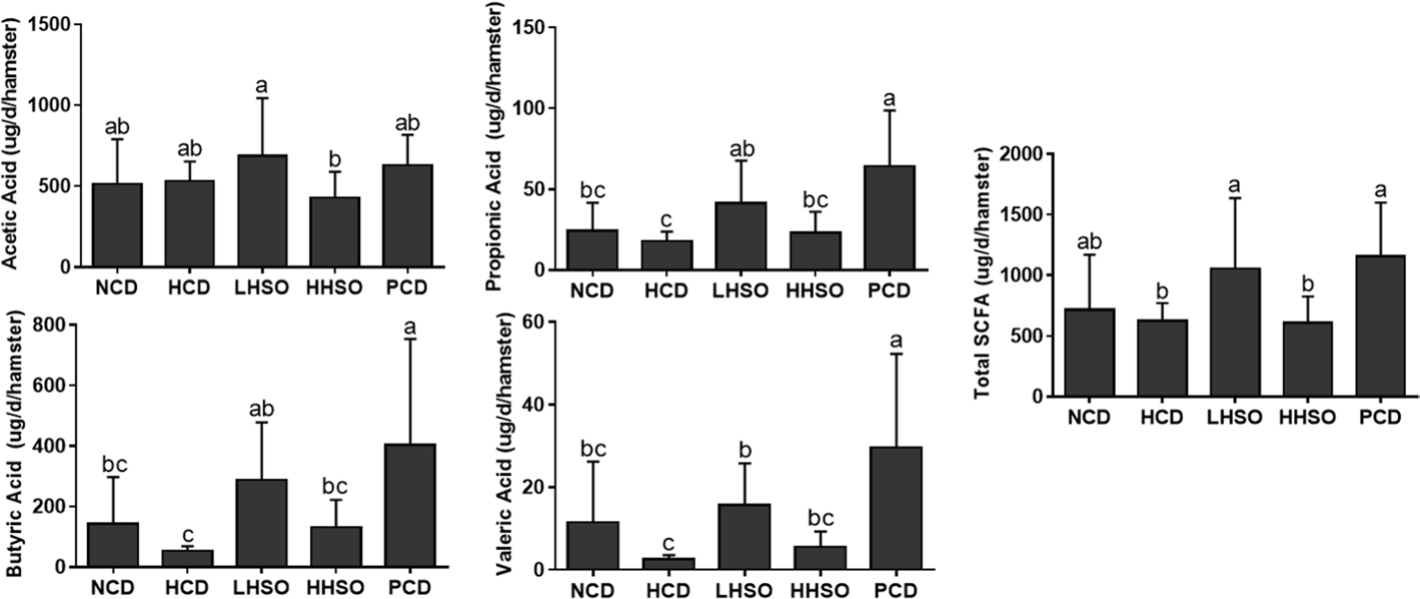
Changes in propionic acid, acetic acid, butyric acid, valeric acid and total short-chain fatty acid in hamsters fed one of the five diets: non-cholesterol diet (NCD); 0.15% cholesterol diet (HCD); hawthorn seed oil substituting 50% lard (LHSO); hawthorn seed oil substituting 100% lard (HHSO), and HCD with 0.5% cholestyramine (PCD). Data is expressed as mean ± S.D., where different letters in superscript indicate statistical difference, *p* < 0.05

Acetic, propionic, butyric and valeric acids are the four SCFAs found in fecal samples. PCD diet increased total fecal SCFAs by 83.9% compared with HCD diet. LHSO could markedly promote the production of acetate (by 28.9%), butyrate (by 431.4%), propionate (by 131.8%) and total fecal SCFAs (by 67.72%) excretion, compared with HCD (Fig. 5). However, HHSO did not have any significant effect on production of fecal SCFAs.

### Effects of HSO on mRNA and protein abundance

**Fig. 6:**
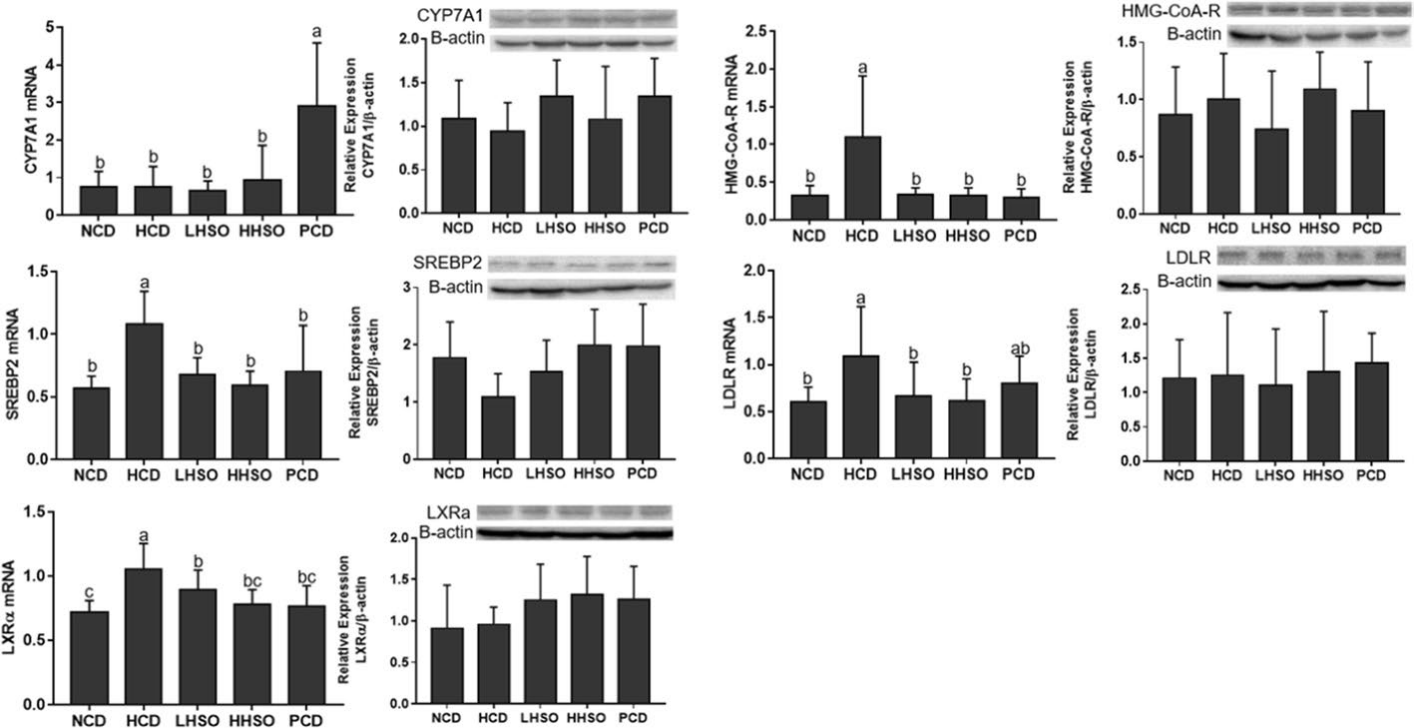
mRNA and protein levels of 3-Hydroxy-3-Methylglutaryl-CoA Reductase (HMG-CoA-R), Sterol regulatory element-binding protein 2 (SREBP2), LDL receptor (LDLR), liver X receptor-alpha (LXRα) and cholesterol-7α-hydroxylase (CYP7A1) in liver of hamsters fed one of the five diets: non-cholesterol diet (NCD); 0.15% cholesterol diet (HCD); hawthorn seed oil substituting 50% lard (LHSO); hawthorn seed oil substituting 100% lard (HHSO), and HCD with 0.5% cholestyramine (PCD). Data is expressed as mean ± S.D., where different letters in superscript indicate statistical difference, *p* < 0.05

**Fig. 7:**
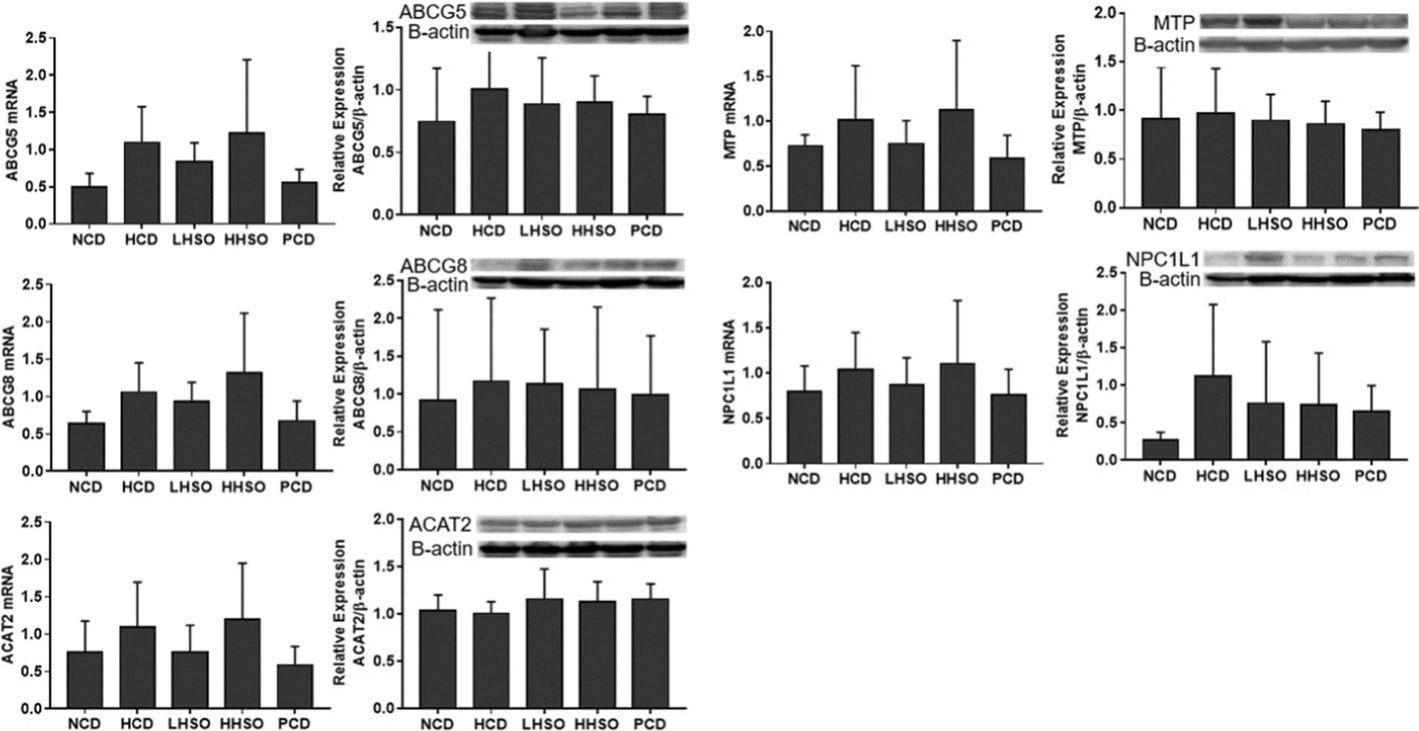
mRNA and protein levels of Niemann-Pick C1-Like 1 (NPC1L1), ATP Binding Cassette Subfamily G Member 5 and 8 (ABCG5/8), Acetyl-CoA Acetyltransferase 2 (ACAT2), and microsomal triacylglycerol transport protein (MTP) in intestine of hamsters fed one of the five diets: non-cholesterol diet (NCD); 0.15% cholesterol diet (HCD); hawthorn seed oil substituting 50% lard (LHSO); hawthorn seed oil substituting 100% lard (HHSO), and HCD with 0.5% cholestyramine (PCD). Data is expressed as mean ± S.D., where different letters in superscript indicate statistical difference, *p* < 0.05

Results from RT-PCR and western blotting indicated that HSO down-regulated mRNA expressions of SREBP2, HMG-CoA-R, LDLR and LXRα in the liver. However, protein quantity in the two HSO experimental diets were not significantly different from that of HCD. Feeding HSO did not alter the gene expression of CYP7A1, whereas PCD feeding up-regulated its expression relative to HCD (Fig. 6). Changes to the genes and protein abundance of the intestinal NPC1L1, ABCG5/8, MTP and ACAT2 were statistically insignificant across groups after feeding (Fig. 7).

### Effects of HSO on gut microbiota diversity

**Fig. 8:**
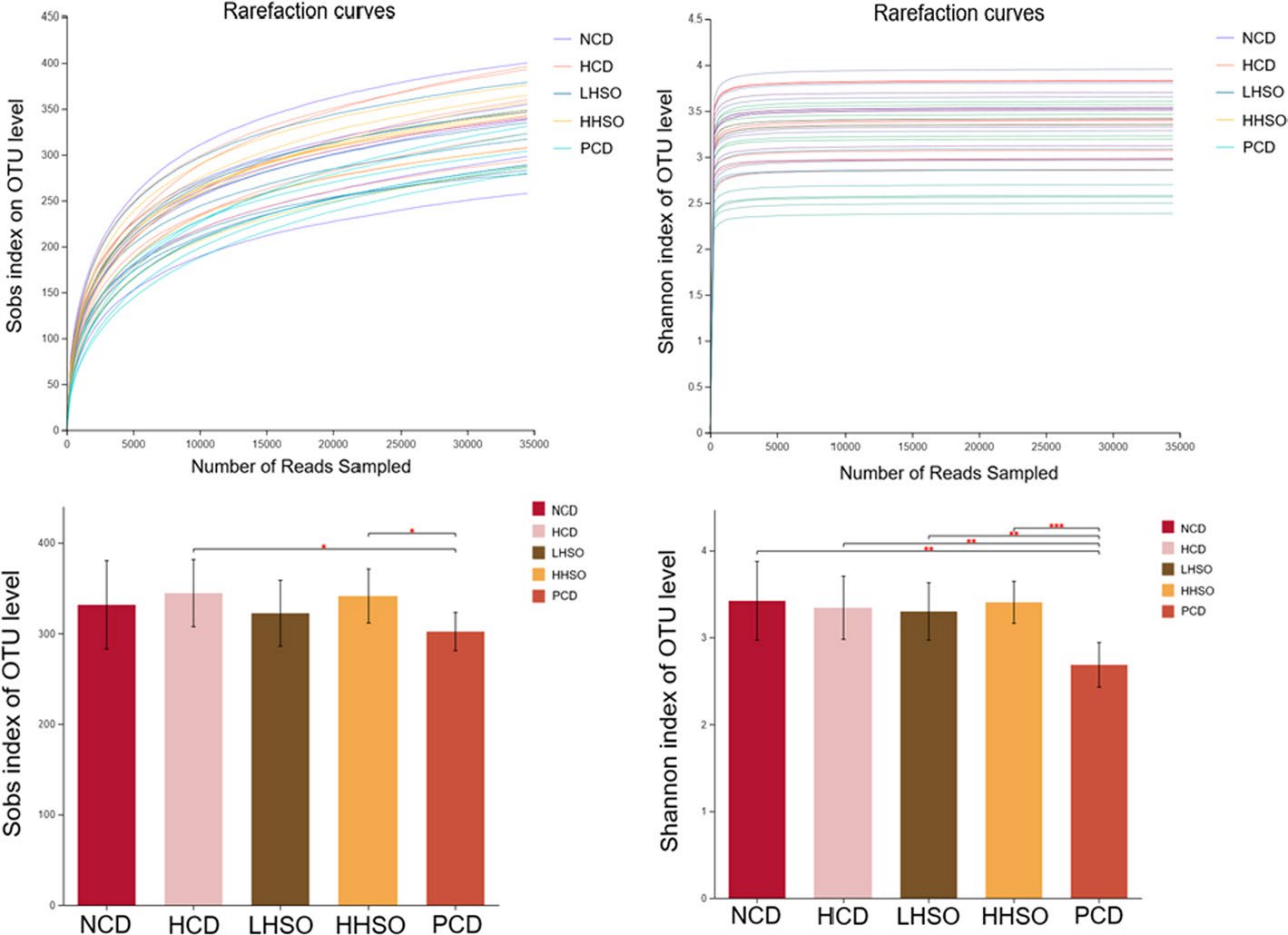
Rarefaction curves and alpha diversity index, representing community richness (Sobs) and diversity (Shannon) of hamsters fed one of the five diets: non-cholesterol diet (NCD); 0.15% cholesterol diet (HCD); hawthorn seed oil substituting 50% lard (LHSO); hawthorn seed oil substituting 100% lard (HHSO), and HCD with 0.5% cholestyramine (PCD)

**Fig. 9:**
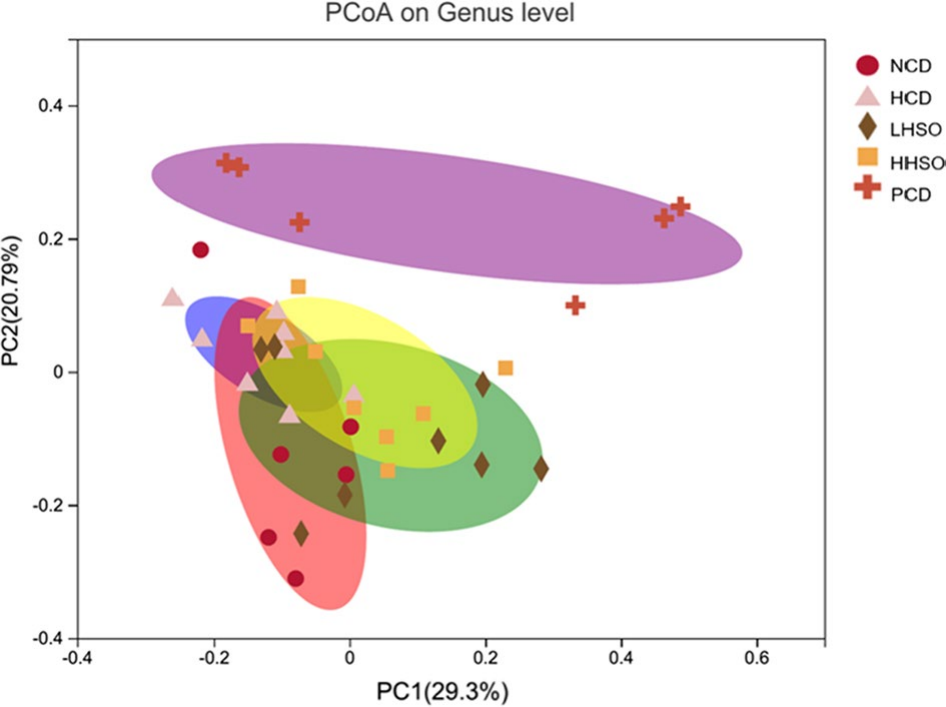
Beta diversity analysis by unweighted UniFrac principle coordinate analysis (PCoA) plot on genus level in hamsters fed one of the five diets: non-cholesterol diet (NCD); 0.15% cholesterol diet (HCD); hawthorn seed oil substituting 50% lard (LHSO); hawthorn seed oil substituting 100% lard (HHSO), and HCD with 0.5% cholestyramine (PCD)

Rarefaction curves were constructed to ensure sufficient sampling was chosen for analysis of alpha-diversity (Fig. 8). Using Shannon index to represent community diversity, and Sobs index for community richness, analyses on the OTU level indicated no significant changes to gut alpha-diversity between HSO groups and HCD (Fig. 8). Beta-diversity PCoA showed genus grouping of PCD was most different with HCD cluster (Fig. 9).

### Effects of HSO on gut microbiota composition

**Fig. 10:**
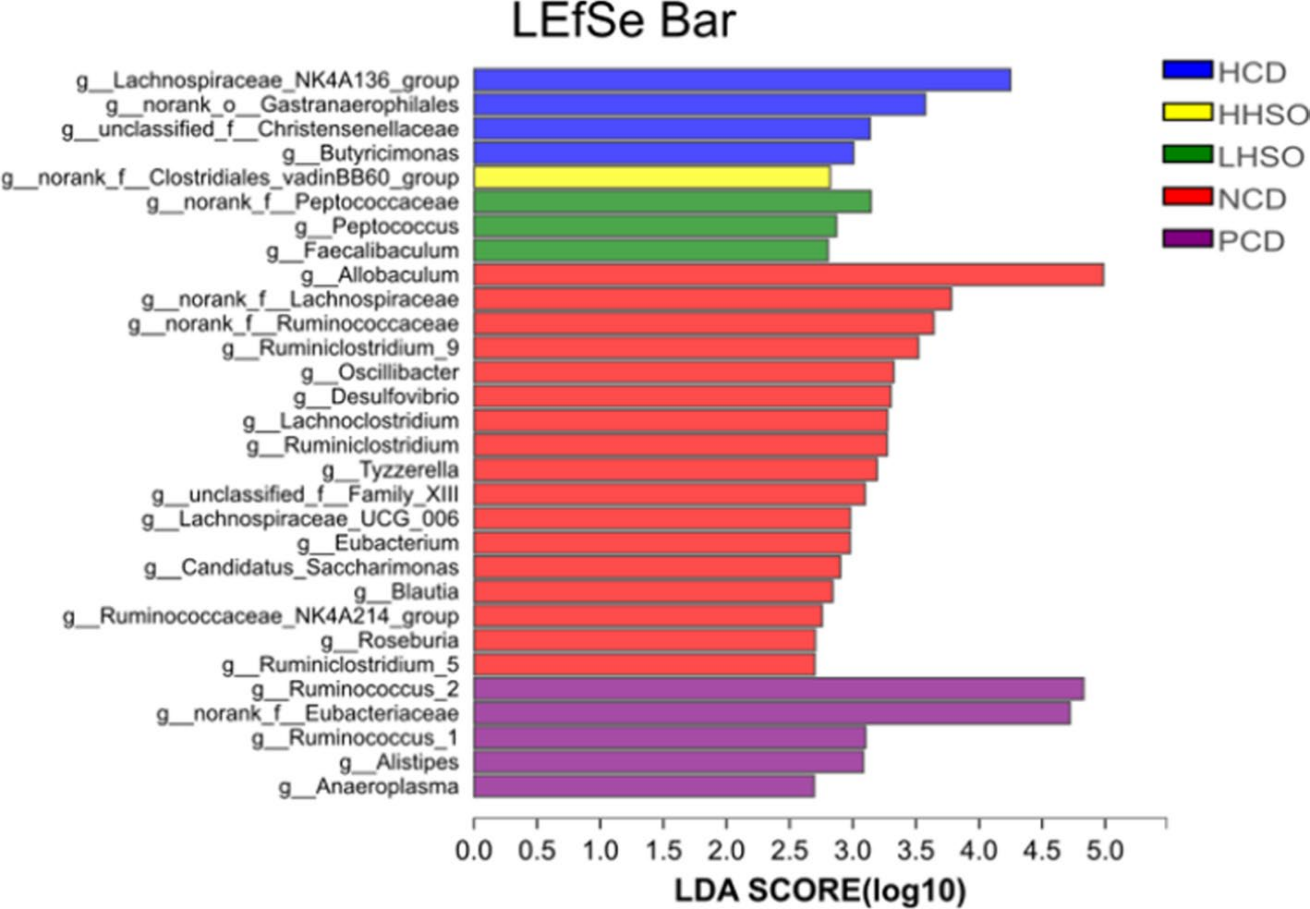
Linear discriminant analysis (LDA) Effect Size (LEfSe) of key genera changes at LDA threshold ≥ 2.5 in hamsters fed one of the five diets: non-cholesterol diet (NCD); 0.15% cholesterol diet (HCD); hawthorn seed oil substituting 50% lard (LHSO); hawthorn seed oil substituting 100% lard (HHSO), and HCD with 0.5% cholestyramine (PCD)

**Fig. 11:**
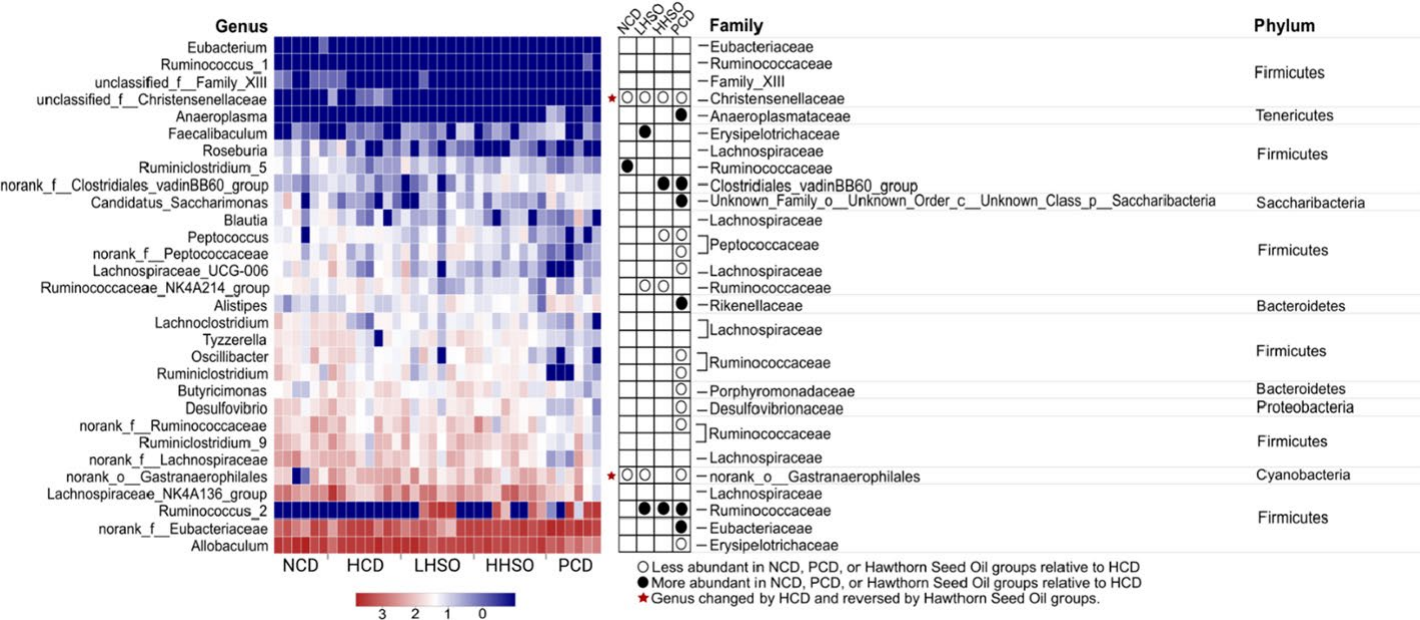
Relative abundance heatmap of 30 specific genera across diet groups. Hamsters were fed one of the five diets: non-cholesterol diet (NCD); 0.15% cholesterol diet (HCD); hawthorn seed oil substituting 50% lard (LHSO); hawthorn seed oil substituting 100% lard (HHSO), and HCD with 0.5% cholestyramine (PCD). Red star represents genera whose abundance was changed by HCD and reversed by Hawthorn Seed Oil groups. Wilcoxon rank-sum test was applied to compute statistical significance, *p* < 0.05. Taxonomy information (phylum, family) is provided on the right

At ≥ 2.5 threshold on LDA score, gut microbiota composition at genus level indicated that 30 main bacterial genus were significantly affected as a result of dietary treatments (Fig. 10). Represented by the community abundance heatmap, LHSO specifically decreased the following genera compared with HCD: *unclassified_f__Christensenellaceae*, *Ruminococcaceae_NK4A214_group*, and *norank_o_Gastranaerophilales*, while increasing abundance of *Faecalibaculum*, and *Ruminococcus_2*. Similarly, HHSO also decreased *unclassified_f__Christensenellaceae* and *Ruminococcaceae_NK4A214_group*, as well as *Peptococcus*. Supplementation of HHSO also increased *norank_f__Clostridiales_vadinBB60_group* and *Ruminococcus_2*. Supplementation of HSO at different doses could reverse HCD-induced changes to *unclassified_f__Christensenellaceae* and *norank_o__Gastranaerophilales* abundance (Fig. 11).

### Correlation analysis on microbial changes and selected parameters

**Fig. 12:**
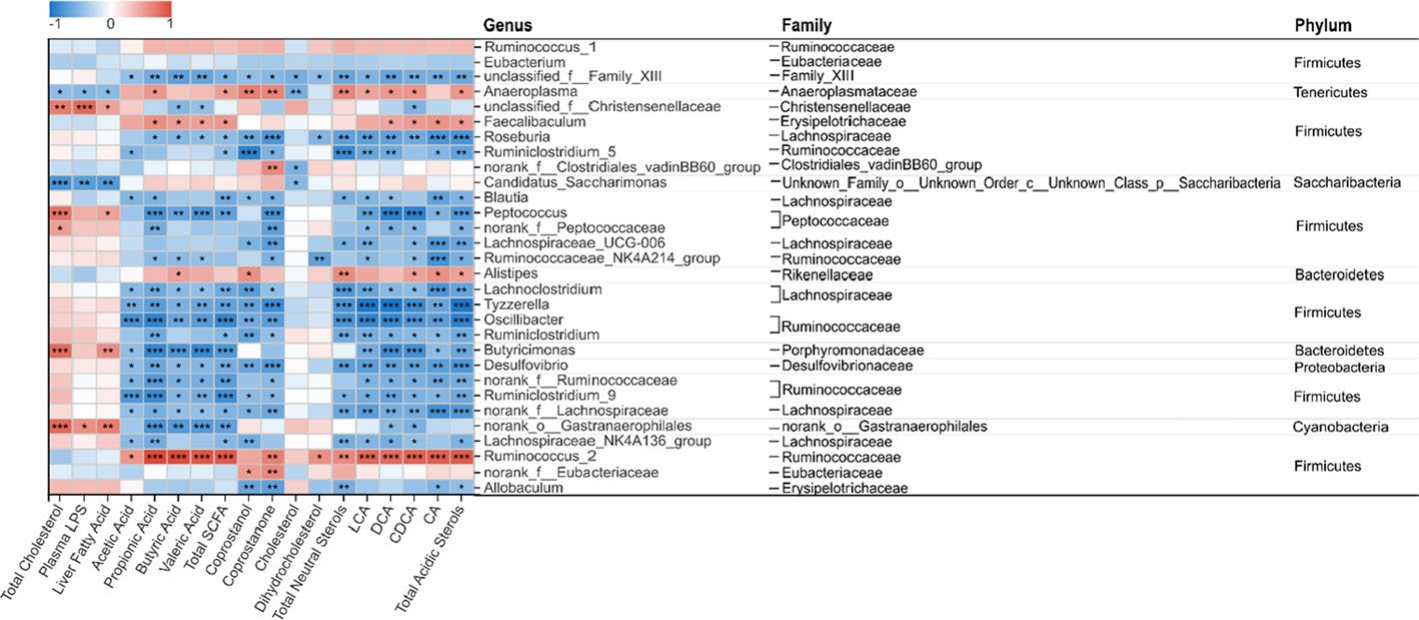
Spearman’s correlation heatmap between 30 specific genera and metabolites related to cholesterol metabolism and other key indices. Hamsters were fed one of the five diets: non-cholesterol diet (NCD); 0.15% cholesterol diet (HCD); hawthorn seed oil substituting 50% lard (LHSO); hawthorn seed oil substituting 100% lard (HHSO), and HCD with 0.5% cholestyramine (PCD). Correlation significance is indicated by asterisks (“*” by *p* < 0.05, “**” by *p* < 0.01, and “***” by *p* < 0.001). Taxonomy information (phylum, family) is provided on the right

Correlation between parameters related to cholesterol metabolism with bacterial genus alterations were conducted with Spearman’s correlation analysis. *Unclassified_f__Christensenellaceae*, *Peptococcus* and *norank_o_Gastranaerophilales* were found to be positively correlated with plasma TC and liver lipids*.* LPS in the blood were similarly correlated with *unclassified_f__Christensenellaceae* and *norank_o_Gastranaerophilales*. Total fecal SCFA amount were directly proportional with *Ruminococcus_2* (including acetate, propionate, butyrate and valerate) as well as *Faecalibaculum* (with propionate, butyrate and valerate). *Norank_o_Gastranaerophilales* and *Peptococcus,* on the other hand, had an inverse relationship with propionate, butyrate, valerate and total SCFA. As a component of neutral sterols, coprostanone had a positive relationship with *Ruminococcus_2* and a negative association with *Ruminococcaceae_NK4A214_group* and *Peptococcus*. Similarly, total neutral sterols were correlated negatively to *Ruminococcaceae_NK4A214_group,* and positively with *Ruminococcus_2*. Finally, total acidic sterols (including LCA, DCA, CDCA and CA), were found directly proportional with *Ruminococcus_2* and *Faecalibaculum* (DCA, CDCA, and CA). Negative correlation was observed between acidic sterols with *Ruminococcaceae_NK4A214_group* and *Peptococcus* abundance (Fig. 12).

## Discussion

The present study was the first time to study the effects of HSO on cholesterol metabolism. Results clearly demonstrated that HSO at two doses could reduce plasma TC significantly by 15–16% compared with that in HCD group (Table 4). The main source of cholesterol in the body is derived from de novo synthesis involving multiple steps: mevalonate production, isopentenyl phosphate production, squalene synthesis, lanosterol production, and finally, cholesterol formation [30, 31]. The rate-limiting step for cholesterol synthesis is governed largely by an enzyme HMG-CoA-R, a catalyst in mevalonate formation from HMG-CoA [32]. HMG-CoA-R gene is regulated by a transcription factor SREBP2 [33, 34]. The present study found that the hepatic gene expression of both HMG-CoA-R and SREBP2 in hamsters fed two HSO diets were significantly reduced. However, the protein masses of HMG-CoA-R and SREBP2 in two HSO diet groups were statistically not different from that of HCD hamsters.

HSO supplementation could generally increase the amount of fecal acidic sterols (Table 5). However, the present study found that the gene expression of CYP7A1, a rate-limiting enzyme for the conversion of bile acid in the liver, was not significantly affected by HSO supplementation (Fig. 6), suggesting that the increase in excretion of bile acids associated with HSO supplementation was unlikely mediated by hepatic up-regulation of CYP7A1. This led us to investigate if HSO could modulate the gut microbiota, which is another major factor to affect the bile acid excretion and plasma cholesterol [35–37]. Spearman’s correlation analysis of gut microbiota composition showed that bacterial genus *Ruminococcus_2* and *Faecalibaculum* was positively correlated to bile acids, whereas *Ruminococcaceae_NK4A214_group* and *Peptococcus* were negatively correlated with bile acids (Fig. 12). Feeding LHSO diet could increase the presence of bile acid-producing *Faecalibaculum* and HHSO decreased *Peptococcus* abundance. Furthermore, feeding both HSO diets could increase *Ruminococcus_2* and reduce *Ruminococcaceae_NK4A214_group* (Fig. 11). Changes in the abundance of these two genera by HSO supplementation could explain the elevation of fecal acidic sterols.

SCFAs are the products of gut microbial fermentation. Numerous studies have confirmed a strong association between SCFA production and the beneficial regulation of cholesterol, lipid and inflammation [38–40]. The present study found that the genera that are positively correlated with SCFA production were *Ruminococcus_2* and *Faecalibaculum*, whereas that negatively correlated genera included *norank_o_Gastranaerophilales* and *Peptococcus* (Fig. 12). Feeding LHSO increased *Faecalibaculum* and reduced *norank_o__Gastranaerophilales*, whereas HHSO decreased *Peptococcus*. Both LHSO and HHSO diets could elevate SCFA-related *Ruminococcus_2* abundance in the feces (Fig. 11). Similarly, previous studies also found that SCFA-producing *Ruminococcus_2* abundance was selectively increased by dietary acarbose, a drug that can favourably affect serum lipid production [41–43]. *Faecalibaculum* is also classified as a SCFA-generating genera, particularly effective for producing butyric acid [39, 44–46]. Therefore, an increase in *Faecalibaculum* and *Ruminococcus_2* in LHSO group could explain the increase of butyric acid production by 431% compared to HCD (Fig. 5). Studies have shown that SCFAs could also inhibit HMG-CoA-R activity, leading to a decreased cholesterol synthesis [40, 47–49]. It was therefore hypothesized that cholesterol-lowering activity associated with HSO supplementation could also be partially mediated by production of gut-derived SCFAs (Fig. 5).

SCFAs is capable of increasing fatty acid oxidation in the liver while it suppresses the fatty acid synthesis through activation of AMP-activated protein kinase (AMPK) pathway [40, 50]. Correlation analysis indicated that *unclassified_f__Christensenellaceae*, *Peptococcus*, and *norank_o__Gastranaerophilales* were positively linked with liver fatty acid accumulation (Fig. 12). Supplementation of both HSO diets could decrease *unclassified_f__Christensenellaceae*, LHSO further reduced *norank_o__Gastranaerophilales*, and HHSO additionally reduced abundance of *Peptococcus* as well (Fig. 11). Changes in abundance of these genera could lead to production of SCFAs and thus to a decrease in liver lipids in the HHSO group (Fig. 3).

Feeding two HSO diets could significantly reduce pro-inflammatory biomarker LPS in the plasma (Fig. 2). Feeding HCD diet led to an increased abundance of specific inflammatory bacteria, whereas feeding HSO diets could reduce these LPS-correlated genera: *Unclassified_f__Christensenellaceae* and *norank_o__Gastranaerophilales* (Fig. 11). LPS is gut-derived endotoxins that can enter circulation from leaky gut barriers and cause the chronic inflammation, atherosclerosis and CVD pathogenesis [38, 51, 52]. It has been known that SCFAs could mitigate the LPS-induced inflammation and protect the gut barrier integrity [39, 53, 54]. The present findings supported this inverse relationship between SCFAs and LPS. It was clear that the bacteria which showed a positive correlation with LPS were negatively correlation with SCFAs (Fig. 12).

The present study did not find a dose-dependent effect on plasma cholesterol and gut microbiota between LHSO and HHSO diets. These variations could be attributable to differences in a ratio of fatty acid composition between the diets of the two experimental groups. Numerous reports have suggested that dietary fatty acid composition plays an important role in regulating bacteria balance and other gut-derived processes [55–57]. In studies comparing the effects of lard and fish oil on abundance of gut bacteria *Akkermansia*, fat intake with different fatty acid composition yielded the conflicting results [58, 59]. Similarly, a previous study also found that hamsters fed an varying amount of plant seed oils affected gut microbiota and cholesterol metabolism differently [25]. In this connection, it deserves a further investigation why HSO at different intake levels could affect gut microbiota differentially.

## Conclusions

The present study explored the effects of lesser-known HSO on blood cholesterol and gut microbiota. It was found that supplementation of HSO could decrease plasma total cholesterol and inflammation in diet-induced hypercholesterolemia hamsters. Such benefits were most likely mediated by favourably modulating gut microbiota and their metabolites (bile acids, SCFA and LPS). Further studies regarding the effects of different dietary fats and oils with different fatty acid compositions on blood cholesterol and gut microbiota will provide greater insights into the interaction among diets, gut microbiota and host cholesterol metabolism.

## Data Availability

All data generated or analysed during this study are included in this published article.

## Abbreviations

CVD: Cardiovascular disease
HSO: Hawthorn seed oil
SCFA: Short-chain fatty acid
PUFA: Polyunsaturated fatty acid
SFA: Saturated fatty acid
MUFA: Monounsaturated fatty acid
GC: Gas chromatography
FAMEs: Fatty acid methyl esters
TC: Total cholesterol
TG: Triacylglycerol
HDL-C: High-density lipoprotein cholesterol
nHDL-C: Non-HDL-C
LPS: Lipopolysaccharide
RT-PCR: Real-time PCR
LDLR: LDL receptor
LXRα: Liver X receptor-alpha
SREBP2: Sterol regulatory element-binding protein 2
HMG-CoA-R: 3-Hydroxy-3-methylglutaryl-CoA reductase
CYP7A1: Cholesterol-7α-hydroxylase
ACAT2: Acetyl-CoA acetyltransferase 2
NPC1L1: Niemann-pick C1-like 1
MTP: Microsomal triacylglycerol transport protein
ABCG5: ATP binding cassette subfamily G-5
ABCG8: ATP binding cassette subfamily G-8
BCA: Bicinchoninic acid
SDS-PAGE: Sodium dodecyl sulfate polyacrylamide gel electrophoresis
TBST: Tris-buffered saline with Tween-20
ECL: Enhanced chemiluminescence
OUT: Operational taxonomic units
PCoA: Principal coordinates analysis
LDA: Linear discriminant analysis
LEfSe: LDA effect size
ANOVA: Analysis of variance
LSD: Least significant difference
CDCA: Chenodeoxycholic acid
CA: Cholic acid
LCA: Lithocholic acid
DCA: Deoxycholic acid
